# Surgeon Attitudes to Guideline-Concordant Extended Pharmacologic Venous Thromboembolism Prophylaxis after Cancer Surgery Within a Regional Health System: A Qualitative Study

**DOI:** 10.1245/s10434-025-17870-0

**Published:** 2025-07-27

**Authors:** Megan K. Scharner, Shannon Phillips, Nivetha Baskar, Natalie Koren, Maggie Westfal, Thomas Curran

**Affiliations:** 1 Medical University of South Carolina College of Medicine, Charleston, SC USA; 2 Medical University of South Carolina College of Nursing, Charleston, SC USA; 3 Division of Colon and Rectal Surgery, Department of Surgery, Medical University of South Carolina, Charleston, SC USA

## Abstract

**Background:**

While infrequent, venous thromboembolism (VTE) is a significant cause of morbidity and mortality after cancer surgery. Extended pharmacologic VTE prophylaxis (ePPX) decreases VTE risk and is recommended by professional societies. Observational studies have shown limited ePPX utilization, although reasons for non-adherence have not been forthcoming from existing data.

**Objective:**

The aim of this study was to obtain insight toward surgeon practices and attitudes regarding ePPX within a regional health system.

**Methods:**

Semi-structured interviews were conducted with 13 surgeons and 2 advanced practice providers who perform gastrointestinal, urologic, or gynecologic cancer resections. Interviews characterized perceptions of VTE risk, VTE prevention strategies, and an electronic medical record-based decision support tool to improve ePPX utilization. Transcripts were thematically analyzed with conceptual coding.

**Results:**

Thirteen surgeons and two advance-practice providers were interviewed; 5 were female. Six surgeons practice in the community and seven practice in the academic setting. Sixty percent (*n* = 9; 8 academic) of providers utilized ePPX ‘routinely’, with professional society guidelines and medical literature the most cited reasons. Thirty-three percent (*n* = 5; 4 community) of providers utilized ePPX ‘selectively’, with injection medication and surgeon routine the most cited reasons. One community surgeon ‘never’ utilized ePPX. Academic providers were more likely to utilize ePPX than community providers. All providers were open to the electronic medical record decision support tool.

**Conclusions:**

Diverse practice patterns of ePPX were identified across providers, with community surgeons more likely to prescribe ePPX selectively or never. Surgeon education, utilization of an oral medication, and cost mitigation may improve ePPX adherence.

**Supplementary Information:**

The online version contains supplementary material available at 10.1245/s10434-025-17870-0.

While infrequent, venous thromboembolism (VTE) is a significant cause of morbidity and mortality after cancer surgery. The in-hospital VTE incidence following abdominopelvic cancer surgery is estimated to be 1.3%.^1^ It is additionally estimated that the 30- and 90-day incidences of VTE following major abdominopelvic cancer surgery are 1.7% and 2.8%, respectively.^2^ However, VTE following cancer resection has been associated with a 5.3-fold increase in mortality as compared with patients who did not suffer a VTE event. Furthermore, studies demonstrate that up to 46% of 30-day deaths following cancer surgery are attributable to VTE.^1,3^ VTE risk disproportionately affects certain patient populations; those of Black race, with public insurance or lack of insurance, and with a high burden of comorbid illness have demonstrated an increased risk of VTE after cancer surgery.^1^ In the era of enhanced recovery after surgery pathways, nearly one-third of VTE cases following major cancer surgery occur after hospital discharge, with 90-day readmission rates for VTE-related complications at up to 1.7%.^4,5^ Importantly, of patients requiring hospital readmission for VTE after cancer surgery, nearly 10% died in hospital.^5^

Extended pharmacologic VTE prophylaxis (ePPX) following major cancer surgery has been shown to be well tolerated and effective in meta-analyses of randomized controlled trials.^6,7^ These data have formed the basis for recommendations of up to 4 weeks of ePPX following major cancer surgery by major professional societies, including the American College of Chest Physicians, American Society of Clinical Oncology, and the National Comprehensive Cancer Network.^8,9^ Despite these recommendations, adoption of and adherence to these guidelines has been limited to 5–13% in state and national observational studies of patients operated for colorectal and pancreas cancer.^10–12^ An internal audit of adherence to ePPX at the authors’ institution identified 23% adherence to ePPX among patients undergoing resection for gastrointestinal, urologic, and gynecologic malignancy from 2015 to 2021. However, the low ePPX utilization in regional and national observational trials is in contrast with survey studies among specialist surgeons, which suggest that surgeons have high levels of awareness and adherence to ePPX guidelines.^13–16^

An improved understanding of the reasons for limited guideline adherence among surgeons may be leveraged to improve utilization of ePPX, which in turn could decrease the rates of postoperative VTE and subsequent morbidity and mortality for patients undergoing cancer surgery. Electronic medical record clinical decision support systems (EMR-CDSS) have been shown to improve adherence to inpatient pharmacologic VTE prophylaxis.^17^ The authors have initiated a stepped wedge randomized controlled trial evaluating the efficacy of an EMR-CDSS for the improvement of guideline-concordant ePPX within a regional health system (NCT06451003). As part of the pre-trial qualitative assessment to refine the intervention, we aimed to obtain insight toward ePPX practices and attitudes among a diverse group of surgeons within a regional health system, as well as to characterize surgeon perceived feasibility of an EMR-CDSS.

## Methods

This study applies a qualitative descriptive design.^18^ Purposive sampling was applied to identify potential participants. Participants sought for inclusion in this study were surgeons and/or surgical advanced practice providers (APPs) who treat abdominopelvic malignancy as a routine part of their clinical practice. Potential participants worked in a regional health network that includes an academic teaching hospital with a National Cancer Institute-designated cancer center, as well as rural and urban community hospitals within the state. Potential participants were approached by a member of the study team via phone and email to explain the study and determine interest in participation. If interested, potential participants were screened for eligibility, and interviews were scheduled with those who screened eligible. The initial sample size was determined based on the principles of information power and was estimated to be up to 15 participants.^19^

Prior to conducting interviews, a semi-structured interview guide with open-ended questions and prompts was developed by the study team. This interview guide is available in electronic supplementary Appendix S1. Questions were designed to explore preferred strategies, barriers, and attitudes regarding perioperative VTE and ePPx, and were informed by the Theoretical Domains Framework paradigm.^20^ In addition to semi-structured interviews, data collection procedures included a visual display of a prototype of an EMR-based decision support tool to improve ePPx utilization. This tool is built into the institution’s EMR and identifies patients who have undergone major surgery for abdominopelvic malignancy in real time, as identified by relevant diagnosis and procedure code dyads. On postoperative day 1, with initiation of the daily progress note, a smart text appears in the ‘Plan’ section of the note. This smart text aids in VTE risk stratification using the Caprini score, and advises providers regarding decision making related to ePPX.^21^ This then carries forward to future progress notes. At the time of discharge, an additional reminder appears during performance of the medication reconciliation. Neither of these things supersede surgical judgment but serve as reminders to the clinical team to provide guideline-concordant ePPX. Participants were then asked questions regarding perceptions of and attitudes towards the tool, and verbally administered the System Usability Scale.^22^ The System Usability Scale was utilized in its original form, which consists of 10 questions, answered on a Likert scale, aimed at assessing subjective assessments of a system’s usability.^22^ The questions are then scored to yield a composite usability score, which ranges from 0 (unusable) to 100 (very usable). Closed-ended questions were included on demographic and clinical practice characteristics. Institutional Review Board approval (#00127734) was obtained at the study site prior to data collection.

After verbal informed consent was obtained, all key informant interviews were conducted via videoconference using the interview guide and additional procedures. Interviews were audio recorded and transcribed verbatim. Transcriptions were reviewed for quality and redacted for identifiable information prior to analysis. Field notes were documented immediately following each interview. Interviews lasted a mean 35 min (range 17–54 min). Data saturation was reached at 15 participants.

Thematic analysis was conducted using Microsoft Word (Microsoft Corporation, Redmond, WA, USA) by two study team members using a hybrid inductive-deductive approach through which data were coded deductively to *a priori* determined codes according to the Theoretical Domains Framework paradigm, and emergent codes were identified inductively.^23^ Coders collaborated weekly during data analysis, which took place between 10 May 2024 and 22 August 2024, to discuss coding, revise coding definitions as needed until consensus was reached, and to determine key themes.

## Results

### Cohort Characteristics

Thirteen surgeons and two APPs were interviewed; 5 were female and 10 were male. Six surgeons practice in the community and seven practice in the academic setting; both APPs practice in the academic setting. The average age of participants was 42.5 years (range 32–58). Three surgeons practice gynecologic oncology, three surgeons practice general surgery, three surgeons practice colorectal surgery, two surgeons practice urology, one surgeon practices surgical oncology, and one surgeon practices hepatobiliary surgery. Further information about participants is characterized in Table 1.

**Table 1:** Cohort description

		All participants [*n* = 15]	Academic practice [*n* = 9]	Community practice [*n* = 6]
Age, years		42.5	42.0	43.4
Years of experience		10.4	9.2	12.2
Male		66.7% (10)	44.4% (4)	100% (6)
Female		33.3% (5)	55.6% (5)	0% (0)
Specialty	Gynecologic oncology	20.0% (3)	33.3% (3)	0.0% (0)
	Surgical oncology	6.7% (1)	11.1% (1)	0.0% (0)
	Urology	13.3% (2)	11.1% (1)	16.7% (1)
	Colorectal surgery	20.0% (3)	11.1% (1)	33.3% (2)
	Hepatobiliary surgery	6.7% (1)	11.1% (1)	0.0% (0)
	General surgery	20.0% (3)	0.0% (0)	50.0% (3)
	Advance practice provider, gastrointestinal surgery	13.3% (2)	22.2% (2)	0.0% (0)

Data are expressed as mean or proportion (n)

### Practice characteristics

Although all interviewed providers work within our regional health system, practice settings and patterns varied. The interviewed providers reported that patients traditionally remain on their surgical service during the postoperative period. However, these services vary by practice location, with residents and/or APPs participating in patient care at some, but not all, locations. When these team members are involved in patient care, the interviewed providers reported that postoperative progress notes and discharge practices are standardized across the team. Although not explicitly addressed in our interview guide, enhanced recovery plans are encouraged and frequently utilized by the interviewed providers, and aim to synergistically decrease VTE risk through early patient mobilization and various other interventions. Finally, the interviewed providers did not cite nursing practice as a modifier to their VTE-related workflow.

### Utilization patterns

**Fig. 1:**
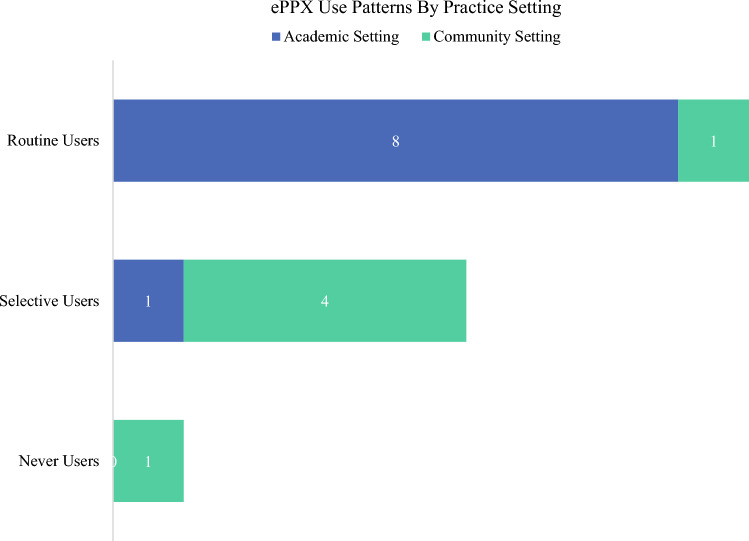
ePPX use by practice setting. *ePPX* extended pharmacologic VTE prophylaxis, *VTE* venous thromboembolism

Sixty percent (*n* = 9; 8 academic) of providers utilized ePPx ‘routinely’, with professional society guidelines and medical literature the most cited reasons. Thirty-three percent (*n* = 5; 4 community) of providers utilized ePPx ‘selectively’, with injection medication administration and surgeon routine/training the most cited reasons. One community surgeon ‘never’ utilized ePPx. These use patterns, categorized by provider practice setting, are described in Fig. 1. Academic providers were more likely to utilize extended VTE prophylaxis than community providers.

### Extended pharmacologic VTE prophylaxis (ePPX) facilitators

Two themes were identified as facilitators to VTE ePPX, the first of which was ‘guidelines and evidence from major societies’. This theme was defined by provider VTE ePPX, as motivated by practice guidelines from their respective organizations, randomized control trials, and personal interpretation of the literature. Comments from 67% (*n* = 10) of providers are represented in this theme. For example, Provider 1 stated: “our patients … standardly get extended prophylaxis because that’s always been the guideline”. Additionally, Provider 2 cited “at least one other randomized control trial that showed benefit [of ePPX] in cancer patients”. Additional characterization and examples of this facilitating theme can be found in Table 2. The second theme found to facilitate VTE ePPX was “surgeon experience and patient outcomes”, which served as an ePPX facilitator for 43% (*n* = 7) of participants. This theme was defined by provider experience of patient outcomes as a facilitating factor for ePPX use. For example, Provider 8 stated: “I think we all have had patients who’ve had terrible outcomes from getting a blood clot”, and Provider 1 stated: “I've had a patient that has died in the perioperative period from a VTE and so I do put it as a priority to prevent that even though the risk is overall small”. These personal anecdotes and outcomes motivated interviewees to prevent VTE events. Further explanation of this theme is outlined in Table 2.

**Table 2:** Facilitators and barriers to ePPx themes

Theme	Theme overview	Frequency proportion (*n*)	Illustrative quote
*Facilitators*			
Guidelines and evidence	Society-based evidence and guidelines support VTE prophylaxis	67% (10)	“After [the patients]^14^ leave the hospital, most commonly they’re discharged on Eliquis because ... the literature that we have has good data to support that.” (Provider 8)
Surgeon experience and patient outcomes	Surgeon experiences and anecdotal patient outcomes may influence their VTE prophylaxis routines	43% (7)	“I can think of 1 patient who almost certainly died of a DVT ... maybe a week or two after discharge, who refused all heparin. All everything.” (Provider 7)
*Barriers*			
Patient comfort and fear	VTE prophylaxis route of administration or adverse effects cause patient fear, which is prohibitive to prophylaxis	60% (9)	“I think in general Lovenox and heparin injections are challenging for some of our patients to do.” (Provider 8)
Cost	Medication cost is prohibitive to patient compliance with VTE prophylaxis	47% (7)	“Lovenox is sometimes cost prohibitive.” (Provider 1)
Surgeon training/routine	Surgeon mentors, training, and length of practice can serve as barriers to VTE prophylaxis	40% (6)	“95% of my practice is driven by mentorship.” (Provider 13)

ePPX extended pharmacologic VTE prophylaxis, VTE venous thromboembolism, DVT deep vein thrombosis

### ePPX barriers

Three themes were identified as barriers to VTE ePPX. The first theme found to serve as a barrier to VTE ePPX was ‘patient comfort and fear’, which was defined as provider hesitancy towards VTE ePPX, as motivated by perceived patient fear of ePPX mode of administration or adverse effects. Sixty percent (*n* = 9) of interviewees cited ePPX route of administration, with emphasis on injection medication, or adverse effects as drivers of patient fear, which is prohibitive to guideline-concordant prophylaxis. Provider 1 stated: “I had a knee surgery. I had to give myself the same injections … they’re not actually fun”. Additionally, when prompted to describe barriers to ePPX, Provider 10 described “a few times [the patients] are saying they’re too concerned about a bleeding risk” as their experience. Further information about this theme is detailed in Table 2. The second theme serving as a barrier to VTE ePPX was ‘cost’, which was defined as provider hesitancy towards VTE ePPX, motivated by perceived patient cost or financial barriers to accessing prophylactic medications. Forty-seven percent (*n* = 7) of interviewees cited cost-prohibitive medications as a barrier to ePPX. Provider 3 clearly stated “the biggest issue would be the cost” when asked about the primary barrier to VTE ePPX. Mitigating factors, cited by multiple providers, to this barrier included case management and social work services, and having the patient speak directly with the pharmacy prior to discharge. Additional information about this theme is provided in Table 2. The final theme found to serve as a barrier to VTE ePPX was ‘surgeon training and routine’, which was defined as hesitancy towards VTE ePPX by means of mentorship-driven practice, surgical training, and length of practice. Forty percent (*n* = 6) of interviewees cited this barrier to VTE ePPX. When asked about barriers to prophylaxis, Provider 3 stated “most of my training was done in an era that [VTE ePPX] wasn’t done”. Additional information and an illustrative quote about this theme are available in Table2.

### Clinical decision support system perceptions

**Fig. 2:**
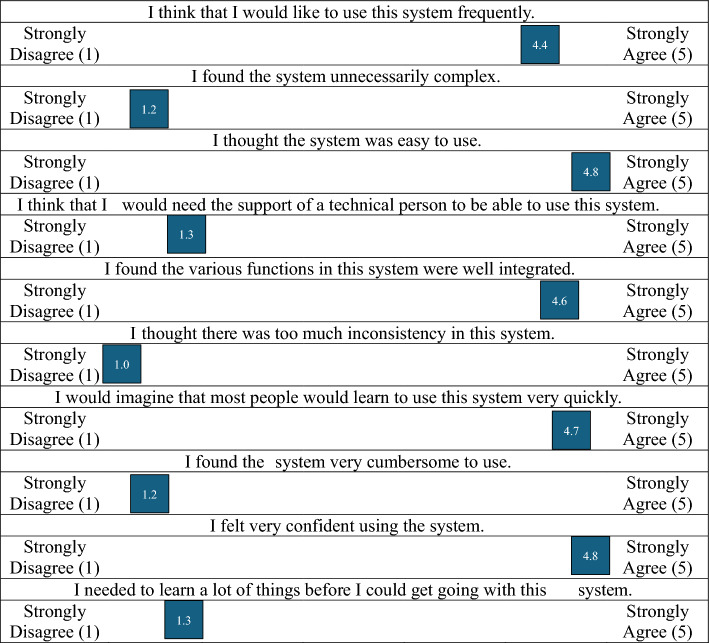
System usability scale^22^

All providers were open to the EMR decision support tool. Qualitative feedback regarding the tool varied among interviewees. Multiple providers suggested that this tool integrated well into current workflow (*n* = 4) and has the potential to help a large number of patients receive guideline-concordant care (*n*= 2). One provider stated that the tool is helpful, “particularly in specialties where [VTE ePPX] guidelines aren’t as specific”. Critiques of the tool included that it may be too gentle or suggestive, rather than requisite (*n *= 4), and that it may increase the length of, and redundancy in, progress notes (*n* = 2). The composite score on the System Usability Scale was 93.25/100, indicating that the EMR decision support tool is very usable.^22^ Average results from each question of the System Usability Scale are characterized in Fig. 2.^22^

## Discussion

In this qualitative study conducted among surgeons from diverse practice settings, we identified mixed attitudes towards ePPX. Community surgeons were more likely to prescribe ePPX selectively or never, and academic surgeons were more likely to routinely prescribe ePPX. Surgeon-perceived barriers to ePPX utilization included cost and presumptions regarding patient comfort with an injection medication. For those surgeons aware of ePPX guidelines, the guidelines were a primary motivator to prescribe ePPX. These findings are revelatory in that they reflect the attitudes of surgeons practicing in both academic and community settings, and second, these findings capture a group of providers that self-report selectively, or never, utilizing ePPX in their clinical practice.

Previous literature reporting on surgeon VTE ePPX utilization in patients undergoing surgery for colorectal or abdominopelvic cancer has almost uniformly been conducted in the academic or tertiary care setting with surgeons who are strongly involved in professional societies and research.^13,14,16,24–26^ In a survey administered to members of the American Society of Colon and Rectal Surgeons, 68% of respondents reported they are both aware of VTE prevention guidelines and use these guidelines in their practice, with 54% of respondents self-identifying as ‘routine users’ of ePPX.^13^ Additionally, in a survey administered to colorectal and hepatobiliary surgeons, 95% of respondents reported adherence to VTE ePPX guidelines.^14^ Themes arising from survey data regarding potential reasons for non-adherence include drug cost, injection mode of administration for low-molecular-weight heparin, and ‘logistical challenges of prescribing’.^14,27^ Additionally, perceived limited evidence has been cited as a reason for limited ePPX adherence, particularly among surgeons who self-report as being in disagreement with VTE ePPX guidelines.^14^ The existing data have also largely been generated from surveys administered to surgical subspecialists or within specialty societies, although the majority of cancer surgery in the United States is performed by non-specialists.^28^

However, our study captures surgeons practicing in both academic (60%, *n* = 9) and community (40%, *n* = 6) settings. To our knowledge, this is the highest proportional representation of community providers in a study assessing ePPX patterns and attitudes for those who treat patients undergoing surgery for cancer, and is important because most cancers in the United States are not surgically treated by specialists.^28^ In a study utilizing the North Carolina Central Cancer Registry, Stitzenberg et al. demonstrated that 48% of cancer surgeries were performed by general surgeons and only 12% were performed by surgical oncologists.^28^ These data suggest that in order to succeed in improving guideline-concordant VTE ePPX, we must focus our efforts on not only academic/specialist providers but also on generalist providers who often practice in the community setting. With this in mind, the proposed EMR-CDSS was broadly acceptable among both academic and community surgeons. One hundred percent (*n* = 12) of surgeons ‘agree’ or ‘strongly agree’ that the system was easy to use, and 92% (*n* = 11) ‘agree’ or ‘strongly agree’ that they would like to use the system frequently. Further perceptions of the EMR-CDSS are available in Fig. 2.

The most frequently cited perceived patient barrier to ePPX in our study was patient comfort and fear, most commonly surrounding injection mode of administration. Sixty percent (*n* = 9) of interviewees cited this as a primary barrier to guideline-concordant care, with primary concerns being patient comfort and patient adherence to injection medication. However, a post hoc analysis of a randomized controlled trial evaluating the safety of apixaban ePPX as compared with enoxaparin ePPX demonstrated excellent patient adherence (84%), with no difference between the apixaban and enoxaparin groups (*p* = 0.89).^29^ Similarly, a large registry of patients undergoing high-risk orthopedic procedures demonstrated excellent adherence to injection ePPX (88%).^30^ Thus, the surgeon barrier of perceived patient non-adherence may be both modifiable and improved with focused education. Moreover, this barrier may become less relevant as data mature regarding the safety and efficacy of direct oral anticoagulants for ePPX. Although only a single randomized controlled trial exists that specifically evaluates the safety and efficacy of apixaban,^31^ multiple centers have adopted direct oral anticoagulants such as apixaban and rivaroxaban.^32,33^ Our participants cited that patients would prefer oral anticoagulants in place of injection medications. Other barriers described by selective users include medication cost and surgeon routine/training. Although medication cost may not be directly modifiable by physicians, the cited mitigators to this barrier, including the utilization of care coordinators/social workers to assist patients with medication costs, as well as patient meetings with pharmacy representatives prior to hospital discharge, may help mitigate some of the perceived barriers. Although surgeon routine and training is not directly or measurably modifiable, knowledge of society guidelines and/or current literature as a facilitator to ePPX may help to alleviate the effects of this barrier. Focused education on society guidelines and current literature within surgical training programs, both at the residency and fellowship level, may help to further facilitate guideline-concordant ePPX. Our description of these barriers may allow for targeted education and intervention for providers, primarily community, who treat the majority of cancers in this country.

### Limitations

This study must be interpreted in the context of its study design/data source. This study was conducted within one regional health system, which may limit transferability to other settings. While inclusive of both academic and community practice settings in various regions of the state, our results require additional contextualization in more diverse practice settings to ensure broad transferability to additional practice settings. However, these findings are novel in that they may shed light on ways in which we can improve guideline-concordant healthcare by allowing investigators to target the specific barriers to ePPX utilization cited by these participants. Our current aim is to integrate these findings through a prospective trial aimed at improving ePPX utilization for patients undergoing abdominopelvic cancer resections.

## Conclusion

Diverse attitudes toward ePPX were identified across providers, with community surgeons more likely to prescribe extended pharmacologic prophylaxis selectively, or never, as compared with academic surgeons who endorsed utilizing ePPX routinely. Surgeons were accepting of an EMR-CDSS, which, if implemented, may improve ePPX utilization/adherence.^17^ Surgeon education of literature and guidelines, utilization of an oral ePPX medication, and medication cost mitigation may improve ePPX adherence. Further research is needed to characterize facilitators and barriers to ePPX in larger cohorts and to trial targeted interventions aimed at improving guideline-concordant ePPX.

## Supplementary Information

Below is the link to the electronic supplementary material.

Supplementary file1 (DOCX 24 kb)
