# Supplementary material for: Surgeon Attitudes to Guideline-Concordant Extended Pharmacologic Venous Thromboembolism Prophylaxis after Cancer Surgery Within a Regional Health System: A Qualitative Study
Source: Ann Surg Oncol. 2025 Jul 27;32(10):7596–603. doi: 10.1245/s10434-025-17870-0 (PMC12454598; doi:10.1245/s10434-025-17870-0)
Supplement: Supplementary file 1 — Supplementary file1 (DOCX 24 kb) [file 10434_2025_17870_MOESM1_ESM.docx]

**Supplemental Appendix S1:** VTE Key Informant Interview Guide

**VTE Key Informant Interview Guide**

- **Session Information**
  - Interview date:
  - Interview team members:
- **Participant Information**
  - Gender:
  - Age:
  - Race (self-identify):
  - Ethnicity (self-identify):
  - Surgical discipline (i.e. General, Urology, Gynecologic Oncology):
  - Practice location:

*Begin recording.*

- **Nature of surgical practice**

***“****The first questions are about the nature of your surgical practice.”*

- - How many years have you been in practice?
  - Do you perform surgery for abdominopelvic cancers and if so, what types of abdominopelvic malignancy do you treat in your practice?
  - What proportion of your practice is dedicated to care for abdominopelvic malignancy and how many of those cases do you perform in an average year?
- **Current venous thromboembolism awareness and prevention**

*“Great, thank you. Now I’d like to ask a few questions about the scope of venous thromboembolism after abdominopelvic cancer surgery and prevention strategies.”*

- - How would you characterize the scope of VTE after cancer surgery in regard to frequency and associated morbidity and mortality?
  - Describe how you discuss VTE with patients preoperatively either in general or as a part of the informed consent process?
  - Across the intraoperative, postoperative inpatient and post-operative outpatient setting, what strategies, if any, do you use to reduce the risk of VTE?
    - Describe any behavioral prevention strategies utilized such as ambulation and physical activity?
    - Describe any mechanical prevention utilized such as compression stockings or sequential compression devices?
    - Describe your utilization of pharmacologic VTE prophylaxis and if so, which drug and for what duration?
  - If you do use post-hospital pharmacologic VTE prophylaxis, what are the reasons for this?
    - Personal experiences?
    - Personal interpretation of available studies?  If so, which studies?
    - Guidelines provided by major professional societies?  If so, which societies?
  - If you do not use post-hospital pharmacologic VTE prophylaxis, what are the reasons for this?
    - Not a priority given competing patient discharge needs?
    - Personal experiences?
    - Personal interpretation of available studies?  If so, which studies?
    - Perception that VTE is too uncommon or not serious?
    - Risk to the patient such as bleeding?
    - Cost to the patient and/or healthcare system?
    - Perception that patients will not adhere to the recommendations?
  - If you selectively use post-hospital pharmacologic VTE prophylaxis, on what basis do you select the patients?
    - Patient or procedural characteristics?
    - Do you use a formal risk stratification tool and if so, which tool?
    - Please explain how practical or logistical constraints play a role such as cost or insurance status?
    - Do you make any changes to your post-hospital pharmacologic extended VTE prophylaxis for patients with CKD?
    - Do you make any changes to your post-hospital pharmacologic extended VTE prophylaxis for patients with social/structural determinants of health affecting their care?
- **Venous thromboembolism prevention guidelines and interventions to improve adherence.**

*“Thanks. Major professional societies advocate consideration of extended VTE prophylaxis following abdominopelvic cancer surgery. A retrospective analysis across GI surgical oncology, urologic surgical oncology and gynecologic surgical oncology at our institution from 2015 to 2021 suggests that we adhere to these guidelines about twenty-three percent of the time. We are developing an intervention to improve our adherence to these guidelines in our system. This intervention includes dedicated education (these interviews, tumor board dedicated education, etc.) and an EMR-based decision support system that will be developed based on the information gathered in this first phase of the study. I’d like to ask you about your postoperative workflow and discharge processes. Then I’d love to hear your feedback on the EMR-based decision support tool.”*

*“We have designed an EMR-based decision support system to improve adherence to guidelines.  This would identify patients who have undergone major surgery for abdominopelvic malignancy in real time. This is identified by procedure code, etc. On postoperative day one, with initiation of the daily progress note, a smart text would appear in the plan.  This smart text will aid in VTE risk stratification and advise regarding decision making related to extended pharmacologic VTE prophylaxis. This could then carry forward to future progress notes. At the time of discharge, an additional reminder would appear during performance of the medication reconciliation. Neither of these things supersede surgical judgment but would provide reminders to the clinical team.”*

- - Based on my description of the EMR-based decision support system, what barriers do you think you and other surgeons would face to using this tool?
  - “What changes and/or resources would need to take place for a tool like this to be successfully used in practice?”
  - We would love to hear your feedback on how this tool may fit into your existing workflow.
    - Please describe the process for daily progress note writing following surgery.
      - Does your team use a common progress note template?
      - Who writes your daily progress notes?
    - Please describe the current process for patient discharge following cancer surgery.
      - Are there standard templates used for post-operative instructions and information?
      - Who discharges your patients?
  - Please give your perception of patient attitude toward extended VTE prophylaxis.
    - Do you think your patients would be accepting of extended VTE prophylaxis?
    - Would agent matter – apixaban vs. enoxaparin?
    - How would dedicated VTE related information help patients to understand the rationale for this medication?

*Surgeon now has the opportunity to trial the EMR CDSS in the EMR trial environment (expected ~5-10 minutes).*

- - Please share any thoughts you may have on the EMR tool and workflow.
    - Should the tool inform user about guidelines? Risk stratification?
    - Would an interactive risk stratification tool be useful?
    - Is the tool too intrusive? Not disruptive enough?
    - Is there anything about the tool you would change?
  - **System Usability Scale**

1. I think that I would like to use this system frequently.

Strongly disagree 1 2 3 4 5 Strongly Agree

2. I found the system unnecessarily complex.

Strongly disagree 1 2 3 4 5 Strongly Agree

3. I thought the system was easy to use.

Strongly disagree 1 2 3 4 5 Strongly Agree

4. I think that I would need the support of a technical person to be able to use this system.

Strongly disagree 1 2 3 4 5 Strongly Agree

5. I found the various functions in this system were well integrated.

Strongly disagree 1 2 3 4 5 Strongly Agree

6. I thought there was too much inconsistency in this system.

Strongly disagree 1 2 3 4 5 Strongly Agree

7. I would imagine that most people would learn to use this system very quickly.

Strongly disagree 1 2 3 4 5 Strongly Agree

8. I found the system very cumbersome to use.

Strongly disagree 1 2 3 4 5 Strongly Agree

9. I felt very confident using the system.

Strongly disagree 1 2 3 4 5 Strongly Agree

10. I needed to learn a lot of things before I could get going with this system.

Strongly disagree 1 2 3 4 5 Strongly Agree

*“Thank you again for taking the time to talk with me today. Is there anything else you can think of that you’d like to share with us?”*

*End interview, end recording.*

- **Post-Interview Thoughts**
  - General comments:
  - Interview guide:
  - Emerging themes:
